# Association of oral pathology, oral microbiology, and oral oncology

**DOI:** 10.3205/dgkh000574

**Published:** 2025-08-19

**Authors:** Divya Sri Lokaranjan, Kamalam Ravi, Susmita Choudhary, Anindita Talukdar, Shilpa Dandekeri, Karthik Shunmugavelu

**Affiliations:** 1 Department of Biochemistry, Tagore Medical College and Hospital, Chennai, India; 2 Department of Biochemistry, Sree Balaji Medical College and Hospital, Chromepet, India; 3 Department of Orthodontics and Dentofacial Orthopaedics, Narsinhbhai Patel Dental College and Hospital, Visnagar, Gujarat, India; 4 Department of Pedodontics and Preventive Dentistry, Regional Dental College, Guwahati, Assam, India; 5 Department of Prosthodontics and Crown and Bridge, Nitte, University AB Shetty Memorial Institute of Dental Sciences, Mangalore, Karnataka, India; 6 Department of Dentistry, PSP Medical College Hospital and Research Institute Tambaram Kanchipuram, Tamil Nadu, India

**Keywords:** candida, oral squamous cell carcinoma, HIV

## Abstract

An ecological community of commensals, symbiotic and pathogenic organisms share our body space. Alterations in the ecologically balanced population of microflora result in dysbiosis and are critical determinants of systemic health and diseases, especially in the context of immunosuppression. The oral microbiome and chronic inflammation may have a role in carcinogenesis.

## Abstract (de)

Die ökologische Gemeinschaft von Kommensalen, symbiotischen und pathogenen Organismen teilt sich unseren Körperraum. Veränderungen in der ökologischen Population der Mikroflora führen zu Dysbiose und sind entscheidende Determinanten für die Gesundheit bzw. für Krankheiten, insbesondere im Fall einer Immunsuppression. Das orale Mikrobiom und chronische Entzündungen können bei der Karzinogenese eine Rolle spielen.

## Introduction

The oral cavity has the second largest and second-most diverse microbiota, with over 700 species of bacteria. It contains bacteria, fungi, viruses and protozoa. The oral cavity, with its various niches, is a complex habitat, e.g., Microbes colonize the hard surfaces of the teeth as well as the soft tissues of the oral mucosa [1]. Recent studies indicate that the oral microbiome has essential functions in maintaining oral and systemic health, and the emergence of 16S rRNA gene next-generation sequencing (NGS) has greatly contributed to revealing the complexity of its bacterial components [2]. 

Few studies have characterized aspects of the oral microbiome that may be related to oral squamous cell carcinoma (OSCC). Features of the oral microbiome associated with OSCC have been explored by comparing OSCC patients with healthy controls, or by comparing tumor sites with the surrounding normal tissue [3]. Pushalkar et al. [4] studied the saliva microbiome of patients with OSCC and proposed its potential use as a diagnostic tool to predict oral cancer. *Candida (C.) auris* has emerged as a multidrug-resistant ascomycete yeast. The saliva microbiome can also contain *C. auris*, which has emerged and is easily transmissible and highly persistent on environmental surfaces [5]. It is associated with high mortalities, persistent candidaemia, inconsistencies in testing results, misidentification and treatment failure. This leads to complications in management and prognosis [6]. 

Thus, the aim of this article is to

- analyze the prevalent microbial population in healthy individuals and patients with oral squamous cell carcinoma using 16s rRNA sequencing and qPCR,
- examine *C. auris* in patients with immune suppression (HIV seropositive), denture wearers (diabetics and non-diabetics) and healthy individuals by using qPCR,
- examine the oral microbiome in HIV-seropositive and HIV-seronegative individuals using 16srRNA sequencing and qPCR.

## Materials and methods

The oral microbiome of the following groups was analysed using 16srRNA sequencing:

- healthy individuals (group A n=10)
- patients with OSCC (group B, n=10)
- HIV-seropositive patients (group C, n=11),
- HIV-seronegative patients denture wearers (group D, n=11).

In groups C and D, the copy number of *C. auris* were additionally determined.

## Results

### Distribution of overall microbial phyla across all groups (A–D)

Proteobacteria was identified as the predominant phylum across all sample groups, contributing up to 39% of the microbial population. This was followed by Firmicutes (22%), Actinobacteria (15%), and Bacteroidetes (12%).

#### Microbial composition in different groups

**Group A (OSCC patients):** The microbial diversity was great, with the genera Bacillus, Buchnera, Caulobacter, Clostridium, Corynebacterium, Desulfotomaculatum, Enterococcus, Flavobacterium, Gemmata, Hymenobacter, Lactobacillus, Listeria, Lysinibacillus, Marinifilum, Ruminococcus, Streptococcus, Streptomyces, and Thermoanaerobacter represented.

**Group B (healthy individuals):** These individuals showed the presence of Bacillus, Enterococcus, Lactobacillus, Massilia, Paenibacillus, and Streptococcus.

**Group C (HIV-seronegative individuals):** A total of 102 species were observed, including Legionella.

**Group D (HIV-seropositive individuals)**: A total of 30 species were detected, including Neisseria.

#### Microbial overlaps and taxa

The following genera were common to both OSCC and healthy individuals: Bacillus, Enterococcus, Lactobacillus, and Streptococcus.

Aphanizomenon, Betaproteobacterium, and Methylococcus were found in both HIV-seropositive and seronegative individuals.

### Functional classification of microbial groups

**Saccharolytic bacteria: **Bacillus, Buchnera, Clostridium, Corynebacterium, Desulfotomaculatum, Enterococcus, Flavobacterium, Gemmata, Hymenobacter, Lactobacillus, Listeria, Ruminococcus, Streptococcus, Streptomyces, and Thermoanaerobacter.

**Aciduric bacteria:** Bacillus, Caulobacter, Clostridium, Corynebacterium, Desulfotomaculatum, Enterococcus, Lactobacillus, Listeria, Lysinibacillus, Ruminococcus, and Streptococcus.

**Aerobic bacteria:** Buchnera, Caulobacter, Clostridium, Corynebacterium, Gemmata, Hymenobacter, Lysinibacillus, and Streptomyces.

**Anaerobic bacteria:** Bacillus, Desulfotomaculatum, Enterococcus, Flavobacterium, Lactobacillus, Listeria, Marinifilum, Ruminococcus, and Streptococcus.

### Site-specific microbial distribution in OSCC patients (group A)

Streptomyces was observed in both alveolus (20%) and tongue (20%). Bacillus and Listeria were exclusively present in alveolar lesions (30%). Streptococcus was the predominant bacterium across all OSCC sites:

- Tongue: 10%
- Buccal mucosa: 20%
- Alveolus: 10%
- Palate: 20%

### Distribution of phyla in the control group (group B)

Proteobacteria was the most prevalent phylum, accounting for 35% of the microbial community in healthy individuals.

### Oral microbiome in HIV-positive and HIV-negative individuals

**HIV-seronegative individuals (group C):** A total of 102 microbial species were detected, including Legionella.

**HIV-seropositive individuals (group D):** A total of 30 microbial species were identified, including Neisseria.

**Common microbes in both HIV-seropositive and -seronegative individuals:** Aphanizomenon, Betaproteobacterium, and Methylococcus.

### Candida (C.) auris copy numbers in different groups

The quantification of *C. auris *showed significant variation across different study groups:

- Denture wearers: Highest average copy number of 548,401.1.
- HIV-seropositive patients: Copy number of 474,966.4.
- Healthy controls: Four out of ten control samples showed no detectable *C. auris*, while the remaining samples had a low average copy number of 9,792.71.

### Anaerobic vs. facultative anaerobic microbial distribution

**In OSCC patients (group A):** Obligate anaerobes comprised 22% of the microbiota.

**In healthy individuals (group B):** Only facultative anaerobes were present.

## Discussion

### Method

16S rRNA sequencing is a very powerful tool for comparative microbiome analysis. The BLAST Basic Local Alignment Search Tool results derived using 16S rRNA, gene DNA sequences was used to identify the evolutionary relationship by a phylogenetic tree. The limitation was that it identifies only the Shine-Dalgarno domain, a domain common in Bacteri and Archaea with an overlap with mitochondrial and chloroplast RNA.

### Results

The oral microbiome is complex. Our study showed that there were differences between the microbiome of OSCC subjects and healthy individuals. The data from this study will help us to identify the species which need to be studied further to ascertain whether they play a part in oral carcinogenesis. 

## Conclusion

Proteobacteria was the most prevalent phylum across all groups, followed by Firmicutes, Actinobacteria, and Bacteroidetes. OSCC patients exhibited a unique microbial profile distinct from healthy individuals, with specific genera present only in OSCC cases. Microbial diversity was significantly lower in HIV-seropositive individuals compared to HIV-seronegative individuals. *C. auris* had the highest prevalence in denture wearers, followed by HIV patients, was minimal in healthy controls, and absent in OSCC patients. Additionally, obligate anaerobes were more dominant in OSCC patients, whereas only facultative anaerobes were found in healthy individuals. These findings contribute additional data on microbial distribution across different health conditions and provide initial clues as to the role of microbial composition in OSCC and HIV as it affects the oral cavity.

## Notes

### Authors’ ORCIDs

- Sri Lokaranjan D:https://orcid.org/0009-0006-1758-8201
- Ravi K: https://orcid.org/0000-0002-9625-3058
- Dandekeri S: https://orcid.org/0000-0001-7814-6067
- Shunmugavelu K: https://orcid.org/0000-0001-7562-8802

### Ethical approval 

Ethical approval obtained

### Funding

None. 

### Competing interests

The authors declare that they have no competing interests.
